# Relationship between adiponectin, TNFα, and SHBG in prepubertal children with obesity

**DOI:** 10.1186/s40348-021-00113-z

**Published:** 2021-03-10

**Authors:** Marta Ramon-Krauel, María Jesús Leal-Witt, Óscar Osorio-Conles, Montse Amat-Bou, Carles Lerin, David M. Selva

**Affiliations:** 1 Endocrinology Department, Institut de Recerca Sant Joan de Déu, Hospital Sant Joan de Déu, 08950 Barcelona, Spain; 2 Diabetes and Metabolism Research Unit, Vall Hebron Institut de Recerca (VHIR), Universitat Autònoma de Barcelona, Pg Vall d’Hebron 119-129, 08035 Barcelona, Spain

**Keywords:** Prepubertal children, Obesity, Sex hormone-binding globulin, Cytokines

## Abstract

**Background:**

Sex hormone-binding globulin (SHBG) levels are low in adult subjects with obesity when compared to normal-weight individuals. Obesity is associated with higher tumor necrosis factor alpha (TNFα) plasma levels and lower adiponectin levels. Moreover, we have recently elucidated the molecular mechanisms by which TNFα and adiponectin regulate hepatic SHBG production.

**Aim:**

The main objective of this study was to assess if the adult associations between TNFα, adiponectin, and SHBG are present in prepubertal children.

**Methods:**

We determined several morphometric and biochemical parameters in normal-weight (*n*=15) and obese prepubertal (*n*=51) children, as well as quantified plasma SHBG, TNFα receptor 1 (TNFα-R1), and adiponectin levels.

**Results:**

Our results showed that prepubertal children with obesity had decreased plasma SHBG levels compared to normal-weight controls (67 nmol/L vs 172 nmol/L). Importantly, SHBG plasma levels correlated significantly (*P* < 0.05) with TNFα (negatively, ßstd= − 0.31) and adiponectin (positively, ßstd= 0.58) suggesting an important role of these two cytokines in determining plasma SHBG levels in prepubertal children.

**Conclusions:**

Our results suggest that plasma adiponectin levels may play a more important role than TNFα in influencing plasma SHBG levels in our prepubertal population with obesity.

## Background

Childhood obesity prevalence has increased at an alarming rate globally, and the International Association for the Study of Obesity estimates that up to 200 million school-aged children are either overweight or obese [1, 2]. This is important because children and adolescents with obesity are at higher risk of obesity during adulthood [3, 4], which is often linked to serious conditions including cardiovascular disease, type 2 diabetes, metabolic syndrome, and several types of cancer [3–8].

It is well-known that adult individuals with obesity show lower plasma sex hormone-binding globulin (SHBG) levels than normal-weight subjects [9, 10]. SHBG is a protein produced and secreted into the circulation by the liver that binds androgens and estrogens with high affinity. In blood, SHBG acts as a carrier of these sex steroids and regulates their bioavailability and access to target tissues and cells [11]. Interestingly, we have previously described that low-grade inflammation occurring in obesity is an important regulator of plasma SHBG levels [12]. Obesity is associated with higher tumor necrosis factor alpha (TNFα) plasma levels and lower adiponectin levels in adults [13, 14]. Moreover, we have recently elucidated the molecular mechanisms by which TNFα and adiponectin regulate hepatic SHBG production [15–17]. Low plasma SHBG levels have been also found in children with obesity; however, the reasons for such reduction need to be yet determined.

In the present work, we aimed to study whether these regulatory mechanisms were already present in prepubertal children. For this purpose, we determined several morphometric and biochemical parameters in normal-weight and prepubertal children with obesity, as well as quantified plasma SHBG, TNFα receptor 1 (TNFα-R1), and adiponectin levels.

## Methods

### Study participants

The study was approved by the Hospital’s Ethic Committee (PIC-21-12). Subsets of data from this cohort have been reported elsewhere [18, 19]. Children with obesity attending the Hospital Sant Joan de Déu, Barcelona (Spain) were recruited between the months of January 2013 and December 2014. Inclusion criteria were children (a) age from 7 to 10 years old; (b) prepubertal, defined as Tanner stage I breast development for girls and testicular volume less than 4 ml in boys; (c) with BMI-SDS for a given age and sex using the World Health Organization (WHO) standards greater than 2 standard deviations for the obesity group, and between −1 and 1 standard deviations for the normal-weight group. Exclusion criteria included any form of endogen obesity, major congenital or chronic disease, drug-induced obesity, use of drugs for weight loss, and involvement in another weight-loss program. All parents signed the informed consent document. Blood samples were obtained from the hospital’s biobank and were available from 15 normal-weight controls and from 51 of the 53 subjects with obesity initially included in the study.

### Physiologic and biochemical measurements

Blood samples were obtained in the morning after an overnight fast (8–10 h of fasting). We measured weight (kg) and height (m) with light clothing in a calibrated scale and rigid stadiometer. Body mass index (BMI) was calculated, and BMI-SDS was obtained by using the “Anthro Plus” software (WHO). Blood pressure was measured in the right arm using an automated system with the appropriate sleeve size for the arm diameter. Waist circumference was determined as middle point between the last rib and iliac crest. Blood samples were taken in tubes containing EDTA, and plasma was immediately separated, aliquoted, and stored at −80 °C until further use. Glucose, insulin, glycated hemoglobin (HbA1c), and the lipid profile (total cholesterol, LDL-cholesterol, HDL-cholesterol, triglycerides) were measured using standard protocols at the Hospital’s clinical laboratory. An oral glucose tolerance test (1.75 g glucose/kg with a maximum of 75 g per subject) was performed in a subset of children with obesity (*n*=47); glucose and insulin levels were measured at 0, 30, 60, 90, and 120 min post-glucose load. The insulinogenic index (IGI) and the whole-body insulin sensitivity index (WBISI) were calculated from the tolerance test data as previously described [20]. Plasma levels for SHBG, adiponectin, and TNFα-R1 were measured by ELISA (R&D Systems, Madrid, Spain).

### Statistical analysis

Data are shown as mean (SD) for normally distributed data, and median [IQR] for non-normally distributed data; groups were compared using two-paired *t*-test or Mann-Whitney *U* test, respectively. Normality was tested with the Shapiro-Wilk W test. SHBG levels were log-transformed to achieve normality before testing correlations. Associations between variables were determined by standard least-square regression, with sex, age, and BMI-SDS as covariates where indicated. Data analysis and calculations were performed in JMP v14.3 (SAS Institute Inc., Cary, NC). A *p* value below 0.05 was considered significant.

## Results

### Circulating SHBG levels are decreased in prepubertal children with obesity

The main clinical and biochemical features of normal-weight (*n*=15) and children with obesity (*n*=51) are displayed in Table 1. All morphometric parameters were significantly elevated in subjects with obesity compared with lean subjects, including weight, BMI-SDS, and waist circumference (Table 1). Regarding the glycemic parameters, no differences were found between lean and obese subjects in glucose and HbA1c levels; however, insulin concentration and HOMA-IR values were significantly increased in children with obesity (Table 1). Regarding the lipid profile, while total cholesterol was not altered, we observed increased LDL-cholesterol and decreased HDL-cholesterol, as well as higher triacylglyceride (TAG) levels in the group with obesity (Table 1). Moreover, subjects with obesity had significantly higher C-reactive protein (CRP) and TNFα-R1 and reduced SHBG and adiponectin plasma levels compared to the normal-weight controls (Table 1).

**Table 1:** Demographic and physiologic characteristics of study subjects. Normally distributed variables are shown by mean (SD) and groups compared by two-tail *t*-test. ^a^Non-normally distributed data are shown by median [IQR] and groups compared by Mann-Whitney *U* test. Bold font indicates *p*<0.0001. *Min*, minimum value; *Max*, maximum value; *BMI-SDS*, BMI standard deviation score; *SP*, systolic pressure; *DP*, diastolic pressure; *ADIPOQ*, adiponectin; *TAG*, triacylglycerides; *LDL-Cho*, low-density lipoprotein cholesterol; *HDL-Cho*, high-density lipoprotein cholesterol; *n.a.*, not applicable

	Normal weight (***n***=15)		Obesity (***n***=51)		
	Mean ± SD	Min-max	Mean ± SD	Min-max	***p*** value
Sex (F/M)	7/8	.	28/23		n.a.
Age (years)	8.4±0.8	(7.3, 10.1)	9.0±1.1	(7.0, 11.0)	**0.048**
Weight (kg)	26.8±2.8	(22.8, 34.8)	53.5±12.6	(32.2, 96.3)	**< 0.001**
BMI-SDS	− 0.01±0.89	(− 1.71, 1.3)	3.41±0.76	(2.04, 5.76)	**< 0.001**
Waist Circ. (cm)	56±3	(50, 60)	83±9	(65, 104)	**< 0.001**
SP (mmHg)	104±7	(94, 116)	113±9	(92, 143)	**0.001**
DP (mmHg)	64±6	(52, 73)	70±7	(54, 83)	**0.003**
SHBG (nmol/L) ^a^	172±24	(115, 218)	67±68	(12, 195)	**< 0.001**
TNFα-R1 (pg/mL)	854±230	(578, 1435)	1084±205	(638, 1573)	**0.001**
CRP (mg/L) ^a^	0.6±2.1	(0.2, 27.4)	3.0±3.2	(0.5, 52.2)	**0.005**
ADIPOQ (pg/mL) ^a^	12380±4739	(6431, 20138)	7057±3778	(1214, 17748)	**< 0.001**
Glycemia (mg/dL)	86±6	(77, 94)	85±8	(70, 113)	0.765
Insulin (mU/L)	3.2±1.6	(0.5, 5.7)	13.6±6.6	(2.1, 33.3)	**<0.001**
HbA1c (%)	5.3±0.2	(5, 5.7)	5.3±0.2	(4.9, 5.8)	0.894
Cholesterol (mg/dL)	160±21	(132, 200)	165±24	(115, 214)	0.554
TAG (mg/dL) ^a^	42±17	(29, 70)	70±41	(39, 183)	**<0.001**
LDL-Cho (mg/dL)	89±19	(67, 132)	104±23	(63, 150)	**0.036**
HDL-Cho (mg/dL)	62±11	(44, 82)	44±10	(29, 82)	**<0.001**
IGI ^a^			1.27±1.00	(− 0.01, 5.69)	n.a.
WBISI ^a^			3.06±2.93	(1.23, 11.74)	n.a.
HOMA-IR	0.70±0.35	(0.10, 1.25)	2.87±1.47	(0.44, 6.66)	**<0.001**

### SHBG correlations with anthropometric and biochemical parameters in prepubertal children

After analyzing all the measured parameters in our study population, our results showed that SHBG levels were negatively correlated to BMI-SDS, waist circumference, insulin, HOMA-IR, TAG, TNFα-R1, and CRP plasma levels adjusting for sex and age. In addition, plasma SHBG levels correlated significantly and positively with adiponectin and HDL plasma levels (Table 2). No correlation was found between SHBG and glucose, HbA1c, total cholesterol, or LDL levels (Table 2). Interestingly, while further adjustment for BMI-SDS did not substantially modify SHBG correlations with insulin, HOMA-IR, and adiponectin, its association with inflammation markers TNFα-R1 and CRP plasma levels was completely lost (Table 2).

**Table 2:** Correlations between SHBG levels and anthropometric and physiologic parameters in all subjects. Multivariate linear regression was applied to determine correlations between SHBG levels and other variables in all subjects. Bold font indicates *P* < 0.05. Model 1 adjusted for sex and age. Model 2 adjusted for sex, age, and BMI-SDS. *ßstd*, standardized beta coefficient from the regression model; *BM-SDS*, BMI standard deviation score; *ADIPOQ*, adiponectin; *TAG*, triacylglycerides; *LDL-Cho*, low-density lipoprotein cholesterol; *HDL-Cho*, high-density lipoprotein cholesterol; *n.a.*, not applicable

	Model 1	Model 2		
	ßstd	***p*** value	ßstd	***p*** value
BMI-SDS	− **0.65**	**< 0.001**	-	-
Waist circ.	− **0.61**	**< 0.001**	− **0.14**	**0.013**
TNFα-R1	− **0.31**	**0.012**	− 0.02	0.914
log CRP	− **0.31**	**0.022**	− 0.11	0.478
log ADIPOQ	**0.58**	**< 0.001**	**0.57**	**< 0.001**
Glucose	− 0.12	0.355	-0.23	0.173
Insulin	− **0.73**	**< 0.001**	**-0.63**	**< 0.001**
HbA1c	− 0.01	0.923	− 0.02	0.910
HOMA-IR	− **0.72**	**< 0.001**	− **0.63**	**< 0.001**
TAG	− **0.48**	**< 0.001**	− **0.37**	**0.018**
Cholesterol	0.18	0.201	0.18	0.301
LDL-Cho	0.09	0.516	0.21	0.225
HDL-Cho	**0.43**	**0.001**	0.16	0.269

### SHBG plasma level correlations in prepubertal children with obesity

Given that obesity is intimately associated with SHBG levels, insulin resistance, and inflammation markers, we analyzed SHBG correlations with all measured parameters only in prepubertal children with obesity to avoid this confounding factor. Our results showed that SHBG plasma levels correlated significantly and negatively with waist circumference, insulin, and TAG concentration, while they correlated positively with adiponectin (Table 3). No correlation was found between SHBG and BMI-SDS, % fat mass, TNFα-R1, CRP glucose, total cholesterol, LDL, or HDL plasma levels (Table 3). Further adjustment by BMI-SDS did not substantially modify these correlations (Table 3).

**Table 3:** Correlations between SHBG levels and anthropometric and physiologic parameters in subjects with obesity. Multivariate linear regression was applied to determine correlations between SHBG levels and other variables only in subjects with obesity. Bold font indicates *P* < 0.05. Model 1 adjusted for sex and age. Model 2 adjusted for sex, age, and BMI-SDS. *ßstd*, standardized beta coefficient from the regression model; *BMI-SDS*, BMI standard deviation score; *ADIPOQ*, adiponectin; *TAG*, triacylglycerides; *LDL-Cho*, low-density lipoprotein cholesterol; *HDL-Cho*, high-density lipoprotein cholesterol; *n.a.*, not applicable

	Model 1		Model 2	
	ßstd	***p*** value	ßstd	***p*** value
BMI-SDS	− **0.34**	**0.008**	n.a.	n.a.
Waist circ	− **0.36**	**0.001**	− 0.16	0.053
TNFα-R1	− 0.14	0.313	− 0.01	0.926
log CRP	− 0.14	0.324	0.03	0.856
log ADIPOQ	**0.58**	**< 0.001**	**0.57**	**< 0.001**
Glucose	− 0.19	0.198	− 0.27	0.081
Insulin	− **0.64**	**< 0.001**	− **0.62**	**< 0.001**
HbA1c	− 0.03	0.823	0.03	0.840
HOMA-IR	− **0.63**	**< 0.001**	− **0.62**	**< 0.001**
log IGI	− **0.58**	**< 0.001**	− **0.53**	**0.001**
log WBISI	**0.59**	**< 0.001**	**0.57**	**< 0.001**
TAG	− **0.33**	**0.028**	− **0.36**	**0.025**
Cholesterol	0.26	0.090	0.12	0.427
LDL-Cho	0.27	0.076	0.14	0.363
HDL-Cho	0.24	0.114	0.22	0.177

## Discussion

Childhood obesity has become a major health concern in the last decades and has increased its prevalence worldwide, in both developed and developing countries [21–23]. It has been already described that childhood and adolescent obesity is associated with an increase in BMI and excess of adiposity [5, 24] which was also evident in our study since prepubertal children with obesity showed higher BMI-SDS and waist circumference when compared with lean children. This adiposity increase occurring in obesity is also associated with insulin resistance, hypertension, and dyslipidemia, which in turn increase risk of developing atherosclerotic vascular disease and type 2 diabetes [5, 24]. Our results corroborated the latter since obese prepubertal children had higher insulin plasma levels together with an increase in HOMA-IR and higher LDL and TAG plasma levels when compared with lean prepubertal children.

An excess of adiposity may also influence various aspects of pubertal development, such as timing of pubertal initiation and hormonal parameters during puberty. How obesity may perturb several hormonal aspects of puberty is currently under debate; however, it may involve sex hormone-binding globulin (SHBG), a liver-produced glycoprotein that binds sex steroids with high affinity in blood regulating their bioavailability [11]. Interestingly, SHBG plasma levels change through human life-span, remaining high during infancy and decreasing when puberty approaches [25]. It is important to mention that low plasma SHBG levels are found in children with obesity; this is in agreement with our results showing that prepubertal children with obesity had half the plasma SHBG levels present in normal-weight prepubertal children.

Low plasma SHBG levels are also present in adult subjects with obesity [9, 10]. The reason why this may occur has been investigated during the last three decades; initially, it was suggested that hyperinsulinemia was the main factor reducing plasma SHBG levels [26, 27]. In fact, a negative correlation between plasma SHBG and insulin levels has been previously described [28, 29], which we also found in our study. However, insulin as a major factor downregulating SHBG production does not come along with the fact that obese subjects are characterized by insulin resistance [30, 31], and, to the best of our knowledge, the molecular mechanism by which insulin reduces SHBG production has not yet been described. In this regard, we have demonstrated that insulin does not regulate hepatic SHBG production [15, 32]. Nevertheless, we found strong correlations between SHBG and insulin plasma levels when analyzing our study population or children with obesity alone. Furthermore, SHBG was strongly associated with both insulin sensitivity (HOMA-IR and WBISI) and insulin secretory (IGI) indexes in children with obesity.

Accumulated evidence over the last decade points to inflammation as one of the critical processes associated with the development of obesity, insulin resistance, and type 2 diabetes [33, 34]. In fact, obesity is considered a state of chronic low-grade inflammation [35, 36]. This is of importance since we have reported that alterations in proinflammatory and anti-inflammatory cytokines occurring in obesity [37, 38] play an important role in regulating hepatic SHBG production [12, 15–17, 39]. Specifically, using in vitro and in vivo models as well as human samples, we have shown that hepatic SHBG production was reduced by TNFα and increased by adiponectin [15–17]. Mechanistically, we have determined that TNFα decreases SHBG production by reducing the protein levels of hepatocyte nuclear receptor 4 alpha (HNF-4α), the main activator of *SHBG* gene expression [15, 16]. On the other hand, adiponectin increases SHBG production via HNF-4α levels by inhibiting hepatic lipogenesis and stimulating hepatic β-oxidation [17]. Interestingly, we showed that plasma TNFα-R1 levels correlated inversely with plasma SHBG levels in adult obese subjects [15]. Remarkably, prepubertal children with obesity from our study had higher TNFα-R1 plasma levels than lean children, as described previously [40]. Importantly, SHBG plasma levels correlated significantly and negatively with TNFα-R1 plasma levels. However, this correlation was lost after adjusting for BMI-SDS. This is of importance since TNFα levels could be an important factor downregulating SHBG production during early stages of obesity development, but once children reach a certain point of inflammation, TNFα plasma levels may not influence SHBG production.

Plasma adiponectin levels are lower in subjects with obesity, both adult [41] and children, compared to normal-weight controls [42]. These findings were corroborated in our study, since prepubertal children with obesity had lower levels of adiponectin when compared with lean subjects. In addition, it has been previously reported that adiponectin levels were inversely associated with SHBG [43, 44], and this correlation was also found in our prepubertal population. Moreover, this correlation between SHBG and adiponectin was maintained after adjusting for BMI-SDS. More importantly, this SHBG correlation with adiponectin was also found when analyzing only subjects with obesity.

In conclusion, the results obtained in this study show that prepubertal children with obesity had decreased plasma SHBG levels compared to normal-weight controls. Importantly, SHBG plasma levels correlated significantly with TNFα (negatively) and adiponectin (positively), suggesting that adiponectin and TNFα could play a role in determining plasma SHBG levels in prepubertal children. In addition, our results suggest that plasma adiponectin levels may play a more important role than TNFα in influencing plasma SHBG levels in our prepubertal population with obesity.

## Data Availability

The datasets used and analyzed during the current study are available from the corresponding author on reasonable request.

## Abbreviations

BMI-SDS: Body mass index standard deviation score
EDTA: Ethylenediaminetetraacetic acid
ELISA: Enzyme-linked immunosorbent assay
HDL: High-density lipoprotein
HOMA-IR: Homeostasis model assessment of insulin resistance
LDL: Low-density lipoprotein
